# Association of Epstein - Barr virus and breast cancer in Eritrea

**DOI:** 10.1186/s13027-017-0173-2

**Published:** 2017-12-21

**Authors:** Ghimja Fessahaye, Ahmed M. Elhassan, Elwaleed M. Elamin, Ameera A. M. Adam, Anghesom Ghebremedhin, Muntaser E. Ibrahim

**Affiliations:** 1 Department of Molecular Biology, Institute of Endemic Diseases, University of Khartoum, Al-Qasr Street, P.O. Box 102, Khartoum, Sudan; 2 Faculty of Medical Laboratory Sciences, Alzaeim Alazhari University, Khartoum, Sudan; 3 Faculty of Medical Laboratory Sciences, Department of Histopathology and Cytology, Al Neelain University, Khartoum, Sudan; 4 Asmera College of Health Sciences, Asmara, Eritrea

**Keywords:** Eritrea, Breast cancer, Epstein–Barr virus, Polymerase chain reaction, Immunohistochemistry, *in situ* hybridization

## Abstract

**Background:**

The oncogenic potential of Epstein-Barr virus (EBV) in breast cancer is being increasingly recognized. Despite some controversies regarding such role, new evidence is suggesting a culpability of EBV in breast cancer, particularly in Africa where the virus has been originally associated with causation of several solid and hematological malignancies. One example is a report from Sudan implicating EBV as a prime etiologic agent for an aggressive type of breast cancer, where nearly 100% of tumor tissues were shown to carry viral signatures. To get a broader view on such association, other nearby countries should be investigated. The present study aims to determine the prevalence and possible associations of the virus in Eritrean breast cancer patients.

**Methods:**

Detection of EBV genome using primers that target Epstein Barr Encoded RNA (EBER) gene and Latent Membrane Protein-1 (LMP-1) gene sequences was performed by polymerase chain reaction (PCR) on DNA samples extracted from 144 formalin fixed paraffin embedded breast cancer tissues and 63 non-cancerous breast tissue as control group. A subset of PCR positive samples was evaluated for EBER gene expression by in situ hybridization (ISH). Expression of Latent Membrane Protein-2a (LMP2a) was also assessed by immunohistochemistry in a subset of 45 samples.

**Results:**

Based on PCR results, EBV genome signals were detected in a total of 40 samples (27.77%) as compared to controls (*p-value* = 0. 0031) with a higher sensitivity when using the EBER primers. Five out of the 14 samples stained by EBER-ISH 35.71% were positive for the virus indicating the presence of the viral genome within the tumor cells. Of those stained for IHC 7 (15.55%) were positive for LMP2a showing low viral protein frequency.

**Conclusions:**

Based on these findings it can be concluded that EBV in Eritrea is associated with a smaller subset of tumors, unlike neighboring Sudan, thus pointing to possible differences in population predisposition and diseases epidemiology.

## Background

Breast cancer is the most common form of malignancy in women both in developed and the developing countries [1]. In Sub Saharan Africa, although the prevalence is relatively lower compared to that of the western countries, it is characterized by aggressive course and targets more women at a younger age [2]. Breast cancer etiology is not yet entirely known, but its incidence is partially explained by environmental factors including viruses such as Epstein-Barr virus [3]. EBV is closely associated with endemic Burkitt lymphoma in sub-Saharan Africa [4] and more recently reported to be a culprit of breast cancer in Sudan [5]. It is a cosmopolitan γ-herpes virus which infects usually at younger age [6]. Its main target are B lymphocytes but it has a potential to infect epithelial cells as well [7] and is associated with a number of lymphoid and epithelial [8] cancers and thus it is classified as a carcinogenic agent by the International Agency for Research in Cancer [9].

There is conflicting evidence as to the role of EBV in breast cancer [5, 10–25]. The differences are believed to be due to the usage of different techniques, various types of tissue samples, geographical and genetic variation of viral and host genomes, racial and socioeconomic variation of study populations [26–28]. Some authors believe that EBV may play a role in breast cancer oncogenesis not as a primary etiological agent but acts in concert with other co-factors [29]. A meta-analysis study concluded that EBV acts as a cofactor in breast cancer development [30].

In Sudan, where aggressive breast cancer is prevailing, EBV is believed to be associated with this cancer based on viral detection in cancer tissues [5] and on molecular evidence from methylome analysis and expression data published online (bioRxivDoi: 10.1101/03432).

The current study was initiated with the aim of finding out whether EBV is a common etiology of breast cancer in East Africa or whether different countries and populations may present with different pattern.

## Methods

### Patients and tissues

This study comprises formalin fixed paraffin- embedded (FFPE) cancer biopsies from 144 cases of breast carcinoma retrieved from the Department of Histopathology, National Health Laboratory, Ministry of Health, Eritrea during 2013, 2014. The Department is the only of its kind and receives sample from all hospitals within the country. Noncancerous tissue samples from the same directory (*n*=63) were used as a controls. Clinical data including age, tumor type, size and involved lymph node were also collected. Eight 10μ thick sections were cut by a sterile microtome blade from each tumor paraffin blocks for subsequent DNA extraction and then amplification. From a subset (*n*=59) of the study blocks additional 4μm thick sections were cut and put onto positive charged slides (Dako) for the use of immunohistochemistry (IHC) as well as *in situ* hybridization (ISH) assays.

### DNA extraction from FFPE

DNA was extracted from FFPE breast cancer tissues (n=144) and non-cancerous tissues (n= 63) using guanidine chloride for buccal wash method (Black-well laboratory Cambridge, UK) modified to suite for FFPE samples as follows: without dewaxing samples were subject to lyses solution containing 400mMNaCl, 6M guanidine chloride and 300 μl of 7.5% ammonium acetate without proteinase K and heat treated at 98°C for 20 minutes in water bath to reverse formaldehyde modification of the FFPE samples. After cooling10μl (20 mg/ml stock) proteinase K was added and incubated for overnight at 56°C. On day two, second heat treatment was applied by incubating samples at 98 °C for 5 minutes in a water bath. After cooling 10μl proteinase k was added, briefly vertoxed and incubated at 56 °C for overnight. During the whole incubation period samples were put on a shaker at interval for about 30 minutes. Chloroform was then added, the supernatant was collected, and DNA was precipitated by ethanol, dissolved in 100 μl TE storage buffer. The purity and quality of the extracted DNA was analyzed based on absorbance of the extracted DNA at 260 and 280 nm wavelengths using a spectrophotometer (NanoDrop-1000, Thermo Fisher Scientific, and Wilmington, USA).

### PCR amplifications of extracted DNA

Before viral amplification, the DNA quality was checked by amplifying a certain region of the glyceraldehyde-3- phosphate dehydrogenase (GAPDH) gene using primers (forward primer 5‘GGCCTCCAAGGAGTAAGACC3‘and reverse primer: 5‘CCCCTCTTCAAGGGGTCTAC3‘). After validation specific regions of the viral genome were amplified by using two primers: Epstein Barr Encoded RNA (EBER) gene (forward primer 5‘CCCTAGTGGTTTCGGACACA3’ and reverse primer 5‘ACTTGCAAATGCTCTAGGCG3’) [12] and Latent Membrane Protein-1 (LMP-1) gene (forward primer: 5′CCGAAGAGGTTGAAAACAAA 3′ and reverse primer 5′GTGGGGGTCGTCATCATCTC 3′) [5]. DNA from EBV-positive nasopharyngeal carcinoma (NPC) was used as a positive control and nuclease-free distilled water was used as a negative control. Electrophoresis of PCR products were done in 2% ethidium bromide-stained agarose gel in TBE buffer at 90 V for 1 h. DNA bands were visualized by a transilluminator (UV doc, England. DNA ladder 100 base pair (bp) (Fermentas-Russia) was used as indicator of band size.

### EBER RNA in situ hybridization

In order to localize viral transcript within the tumor cells, 14 samples which were positive by PCR in duplicate assay and which have relatively brighter bands in agarose gel were further tested by ISH technique using PNA ISH detection Kit following the manufacturer’s instruction. Briefly, 4 μ thick FFPE tissue sections on positively charges slides (BioGenex, USA) were deparaffinized in xylene, rehydrated in serial graded ethanol washes, digested with proteinase K and then followed by hybridization of EBV- EBER peptide nucleic acid (PNA) Probe/ Fluorescein (Dako) for 90 minutes at 55°C. Detection was accomplished by alkaline phosphatase (AP)-conjugated anti-fluorescein antibodies using nitro blue tetrazolium (NBT)/BromochloroIndoyl phosphate (BCIP) (Dako) as a substrate. Slides were counterstained with hematoxylin (Chem Cruz, Santa Cruz Biotechnology) and mounted with DPX Mountant (Atom Scientific). Positive and negative controls provided by the manufacturer were used. Section from EBV positive nasopharyngeal carcinoma was used as an additional positive control. A case was considered as expressing EBER if the nucleus of a tumor cell stained dark blue or black.

### Immunohistochemistry (IHC) test

Sections of 4 μm thick were cut from 45 paraffin blocks of breast cancer using a microtome and put on coated immunoslides and were de-paraffinized in xylene, rehydrated through series of graded alcohol, submitted to heat retrieval in citrate buffer (pH 6.0) for 40 minutes in water bath. After heating, the slides were allowed to cool to room temperature and washed with phosphate buffered saline (PBS). Endogenous peroxidase activity was blocked with 1% hydrogen peroxide in methanol for 5 minutes blocking serum was used for 1hr in order to block nonspecific immunoreactions. Monoclonal antibodies for LMP2a (Santa Cruz Biotechnology) were applied at a dilution of 1:100 on all tissue sections for overnight at 4°C to evaluate the expression of LMP2a. Detection was performed using Santa Cruz biotechnology envision dual link system according to the manufacturer's instruction. After that, slides were visualized using Santa Cruz liquid DAB. Mayer's hematoxylin was used as a counter stain. As a positive control, we used EBV infected nasopharyngeal carcinoma. In negative controls, the primary antibodies were omitted.

### Statistical analysis

Data were entered and analyzed using the software Statistical Package for Social Sciences version 20 (SPSS, Inc., Chicago, IL, USA). Proportions were compared for significance using the Fisher exact test to determine whether there was any significant difference between the frequency of EBV in the carcinoma and the non-cancerous samples and its relationship with breast cancer. A *p*- value of ≤ 0.05 was considered indicative of a statistically significant difference.

## Results

### Clinicopathological features

During this study breast cancer samples were collected from 144 patients mean age of 51.48 years (range, 19 to 91 years). The mean age of the controls with fibroadenoma was 26 years (range ). Histological tumor types were: ductal carcinoma 96 (66.66%) (Of which 56 (58.33%) were invasive ductal carcinoma), medullary carcinoma 12 (8.33), lobular carcinoma 2(1.38%) and all other types 34 (23.61%). Lymph node involvement was detected in 35 (24.30%) of all cases (Table 1).

**Table 1:** Clinicopathological features of breast cancer samples (*n*=144) from Eritrean patients

Feature	Value
Age (years)	
Mean	51.48
Range	19-91
Histology	
Ductal	96 (66.66%)
Lobular	2 (1.38%)
Medullary	12 (8.33%)
Others	34 (23.61%)
Lymph node involvement	45 (31.25%)

One hundred fourty-four formalin fixed paraffin- embedded (FFPE) tissue sections of breast carcinoma retrieved from the Department of Histopathology, National Health Laboratory, Ministry of Health, Eritrea. The age of the patients ranged from 19 to 91 years with a mean age of 51.48 years. The constitution of the types was: duct carcinoma 66.66% (96/144), lobular carcinoma 1.38% (2/144), medullary 8.33% (12/144) and others 23.61% (34/144). Lymph node was involved in 31.25% (45/144)

**Fig. 1:**
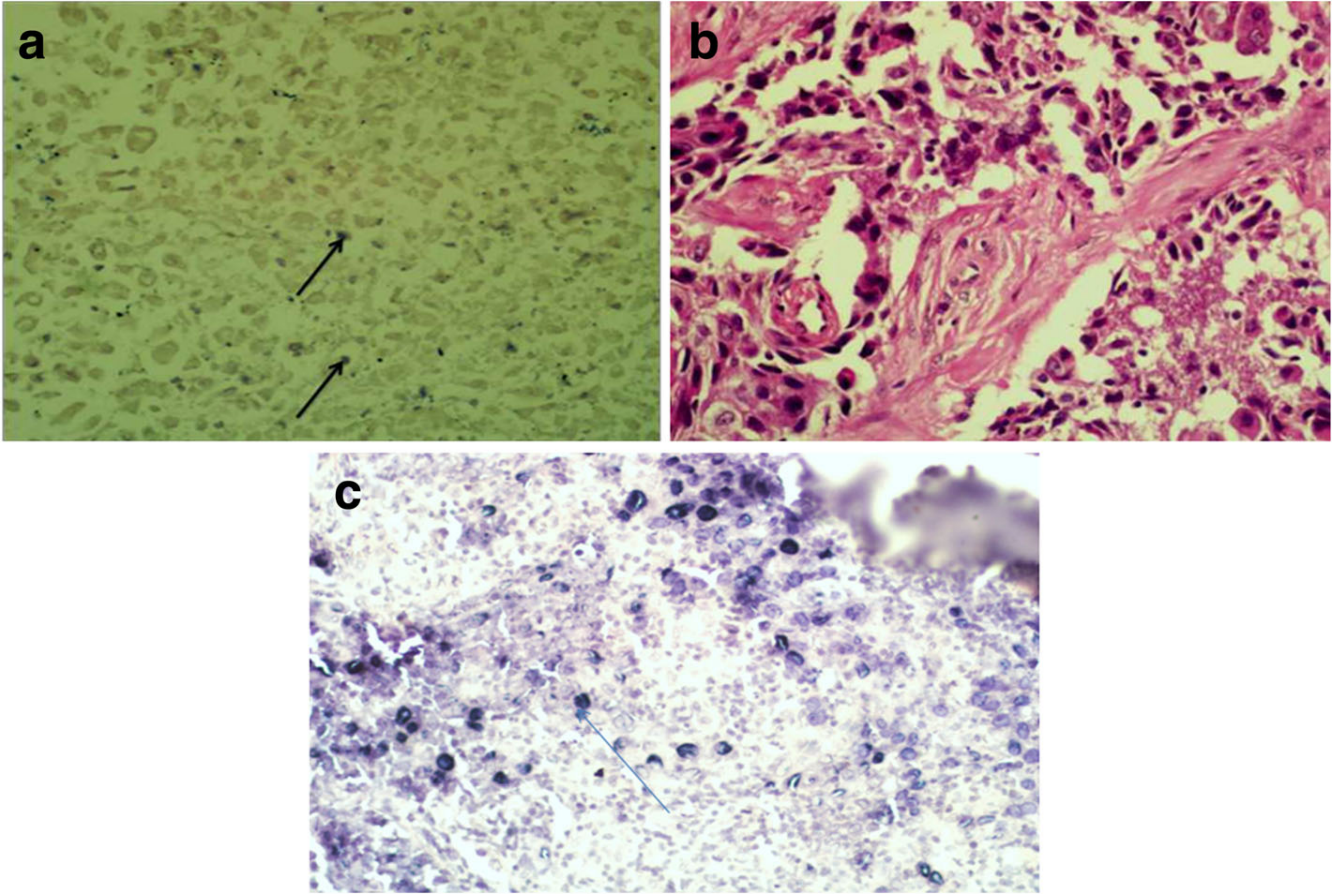
Detection of EBV in poorly differentiated invasive ductal carcinoma tissue section using EBER-ISH nuclear staining. Epstein-Barr virus-encoded RNA-1 (EBER1) in situ hybridization technique was used on those samples which were relatively more positive by PCR (*n* = 14) and signal for viral transcripts was observed in 35.71% (5/14). Figure 1**a** shows a case of poorly differentiated invasive ductal carcinoma exhibiting nuclear positivity indicated by dark staining within malignant cells (arrows). (X 40 objective). Figure. 1**b** is the hematoxyline and eosin staining of the image in Fig. 1**a**. (X 40 objective). Figure 1**c** is a positive control from a case of EBV infected nasopharyngeal carcinoma tissue section showing dark nuclear staining (arrows). (X40 objective)

### Molecular detection of EBV in breast cancer cases

DNA was successfully extracted from 144 FFPE samples of breast carcinoma patients, and from 63 non- cancerous benign tissues samples. The extracted DNA from FFPE breast cancer tissue was successfully amplified using GAPDH primers indicating good quality DNA for further viral detection assay. Amplification fragments of both LMP1 & EBER were detected using PCR. Combining the two markers a total of 40 (27.77%) of 144 breast cancer samples showed faint bands compared to the band formed by the positive control from a case of nasopharyngeal carcinoma. Repeated trial was done for those samples with faint bands and the result was the same as the first trial. Out of the samples with faint band 33(22.91%) were revealed using EBER and 14 (9.72%) using LMP1. As reviewed by Hou and his team, in three previous studies, the detection potential of EBER was higher than that of LMP1 [30]. Six (9.52%) out of 63 control samples showed very faint bands. Statistically, using EBER marker, association between EBV and breast cancer (*P=* 0.0031) was observed compared to the control group but the association employing LMP1 marker was not significant (*P* =0.1563), Fisher's exact test (Table 2).

**Table 2:** Detection of EBV genome in breast cancer samples by PCR using EBER and LMP-1 primers

Primer Breast cancer cases		Noncancerous cases	p-Val*
EBER			
Positive	33	4	0.0031*
Negative	111	59	
LMP1			
Positive	14	2	0.1563
Negative	130	61	

Amplification fragments of both EBER and LMP1 of EBV were detected using PCR. A total of 40 (27.77%) of 144 breast cancer samples showed faint bands compared to the band formed by the positive control from a case of nasopharyngeal carcinoma by either of the markers. Of this faint band 33(22.91%) were revealed using EBER and14 (9.72%) using LMP1and 7(4.86%) were positive by both markers. Out of the 63 control samples 6 by both markers. Were positive (9.52%)showed very faint bands. Statistically, using EBER marker, association between EBV and breast cancer (p-0.0031) was observed compared to the control group but the association employing LMP1 marker was not significant (p-0.1563), Fisher’s exact test

*P value < 0.05 is considered as significant (Fisher’s exact test). Abbreviation: EBER Epstein-Barr virus encoded RNA, LMP1 Latent membrane protein 1

### ISH detection of EBER1 gene transcript

In order to confirm the presence of EBV within the tumor cells, Epstein-Barr virus-encoded RNA-1 (EBER1) *in situ* hybridization technique was used on those cases which were PCR positive in duplicate trials and/or those showing relatively brighter bands on agarose gel electrophoresis and or those which were positive by two markers (*n*=14). Signal for viral transcripts was observed in 35.71% (5/14) indicated by its nuclear localization in the tumor cells. Figure 1a shows positive staining of a case of poorly differentiated invasive ductal carcinoma exhibiting nuclear positivity indicated by dark staining in malignant cell**.** Fig 1b is the hematoxyline and eosin staining of the section in Fig 1a). A tissue section from a case of NPC was used as a positive control (Fig. 1c) and as a negative control a breast cancer section without the primary antibody was employed.

### IHC staining for detection of viral protein (LMP2a)

To assess expression of viral protein, IHC assay using LMP2a antibody was performed on 45 samples and LMP2a was expressed in 15.55% (7/45) of which six (13.33%) were also positive for EBV by PCR.

## Discussion

Three detection methods: PCR. ISH and IHC were employed in the determination of EBV association with breast cancer. PCR results indicated the presence of EBV genome in 27.77% (*n*=144) of breast cancer samples amplified using EBER and LMP1 viral DNA fragments. This finding is in line with a metanalysis study in which about 29.32 % of the patients with breast cancer were infected with the virus [30]. In this study the number of EBV positive samples as well as that of EBV infected cells within positive samples were observed to be quite low as indicated by the faint bands on agarose gel and the scarce infected tumor cells in the ISH stained slides as compared to the positive controls from a case of NPC. PCR amplification was repeated for those samples with faint bands and got similar result. It is reported that as low as 0.00004 EBV genomes per infected cell can be detected using quantitative PCR [20] and Perrigoue and his team define ' EBV positive ' as the majority of tumor cells each to contain at least one copy of EBV DNA [19]. In a similar study low level EBV DNA in about 50% of the study breast cancer samples were detected by PCR [31]. EBV positive tumor infiltrating lymphocytes are believed to give false positive results during PCR assay [23, 32]. To rule out this, EBER-ISH technique which is considered as a gold standard technique for detection of EBV [33] was performed and viral transcripts were detected in 35.71% of the 14 stained samples. Fig. 1a shows one of the positive slides revealing nuclear localization of the viral transcript within the tumor cells, though compared to the positive control from a case of NPC (Fig. 1c) the viral copies seems quite low.

Of interest here, from epidemiological point of view, the frequency difference in Eritrean cases in comparison to neighboring Sudan where a frequency as high as 100% EBV infection in breast cancer has been reported [5]. This variation may be influenced by factors such as geographical and immunological differences and ethnicity [15, 34]. Lopategui and his team in their study in cases of sinonasal undifferentiated carcinomas comparing populations from two different regions suggested that genetic predisposition or environmental cofactors play an important role in determining the strength of the association of malignancy with EBV [35]. Similar to the present work, low copy number of EBV in breast cancer is also reported in other studies though, latently EBV infected cells contain massive EBER viral transcripts [36, 37] as is the case in NPC in which the frequency is 100*%.* Magrath and Bhatia suggested that the presence of EBV genome in only a subset of tumor cells indicates that EBV may infect already formed cancer cell implying its absence in neoplastic clone at the time of malignant transformation, but they do not rule out selective loss of viral genome [32]. Kalkan and his team detected EBV genome in a subset of breast cancer tissues and based on their finding and the findings of other studies, concluded that EBV if it has a role in breast cancer it is only in a limited subset of the cases [38]. In our findings not all PCR positive samples were also positive by ISH. The in concordance between the results of these two assays was previously reported. Richardson and his team reviewed 16 studies that used both ISH and PCR to detect EBV in breast cancer tissue and found out that in nine (56%) of the studies PCR positive cases were negative by ISH [28]. Most of our samples which were EBV-ISH positive were poorly differentiated invasive ductal carcinoma which indicates that the virus may probably have a role in complicating existing tumor. It is known that EBV is usually detected in undifferentiated nasopharyngeal carcinoma [4] and its association with highly invasive breast tumors is also reported [12].

LMP2A is over expressed in 15.55% (*n*=7) of the 45 breast cancer samples stained by immunostaining. This indicates low frequency of viral protein expression in breast cancer samples from Eritrea. This protein was observed to be expressed in about half of the samples from NPC and is mainly localized at the tumor invasive front [39].

EBV is one of the most successful pathogens to establish perfect host pathogen equilibrium and lives latently without too much interfering into the health of humans and according to some authors [40, 41] its carcinogenic effect is the outcome of its coordination with other cofactors. Even in its well established association with lymphomas it is mainly detected in immunodeficiency state and is known as a ubiquitous virus which usually causes cancer in immune suppressed individuals [42]. Some authors believe that, pathogenesis of EBV may be of significance only in the presence of environmental carcinogens or pre-existing epithelial damage [32].

Interestingly, EBV genome was also detected in six (9.52%) out of a total 63 controls (fibroadenomas). One sample was also positive by IHC for LMP2a protein and another one was positive by EBER-ISH. The role of EBV in the pathogenesis of fibroadenoma is suggested in a study performed on immunosuppressed individuals [43]. In another study benign breast tissues were positive for EBV DNA [38] and it is reported to infect non- cancerous gastric epithelium such as atrophic gastric mucosa which may progress to cancer [44, 45]. Further study is required for the role of EBV in non-cancerous breast tissues such as fibroadenomas.

In this study, even if the viral genome was detected within tumor epithelial cells and the low copy number was accepted as having a role in pathophysiology of the disease; majority of our test samples were free of the viral genome. The loss of viral genome during tumor development due to ‘Hit and run’ hypothesis was previously suggested [46] but have doubt as whether this can explain the whole scenario. Thus, in this study, though there seems to be an association of EBV with breast cancer, considering the low number of infected samples and low viral copy number in each infected samples, it is difficult to generalize the viral casual role.

## Conclusion

Based on the present finding which showed very low frequency of EBV in breast cancer tumors as well as low viral copy number within positive tumors, it can be concluded that though there could probably be some sort of association between EBV and breast cancer, its role as prime tumor initiator in breast cancer in Eritrea seems less probable. Further studies are required to determine the role of the few infected cells in carcinogenesis as well as tumor progression and the epidemiological cofactors which impact on EBV association with breast cancer in Eritrea.

## Abbreviations

EBER: Epstein Barr Encoded RNA
EBV: Epstein-Barr virus
FFPE: Formalin fixed paraffin- embedded
GAPDH: Glyceraldehyde-3-phosphate-dehydrogenase
IARC: International Agent for Research in Cancer
IHC: Immunohistochemistry
ISH: In situ hybridization
LMP-1: Latent Membrane Protein-1
LMP2a: Latent Membrane Protein-2a
NPC: Nasopharyngeal carcinoma
PCR: Polymerase chain reaction
